# Chest imaging case atlas

**DOI:** 10.1002/jmrs.16

**Published:** 2013-06-12

**Authors:** Michael Haigherty

**Affiliations:** Department of Medical Imaging, Level 3, Ned Hanlon Building, Royal Brisbane and Women's Hospital Metro North Health Service DistrictCnr Butterfield St and Bowen Bridge Rd, Herston, QLD, 4029, Australia

*Chest Imaging Case Atlas* is the re-titled and greatly anticipated second edition of the acclaimed *Teaching Atlas of Chest Imaging*. It is written by internationally respected authors, Drs. M. S. Parker, M. L. Rosado-de-Christenson, and G. F. Abbott, leading authorities in the realms of chest imaging.

This giant atlas is literally a heavy weight with its 933 A4-sized, semi-gloss, heavy-gauge pages. Bound in a sturdy, robust, yet stylish hard-back presentation, it will be noticed on any bookshelf.

The overall format of the atlas remains the same as the original with its well-structured, straightforward, reader-friendly format.

The atlas is comprised of 13 sections, with the first section providing the reader with a complete, concise, overview of normal thoracic anatomy and the relationships between anatomical components and neighbouring structures. The writers use comprehensible and appropriate medical terminology without the excessive and unnecessary medical jargon that can be found in other medical and academic publications. The overview is greatly enhanced with a combination of clearly labelled artist illustrations, high-quality radiographs, and multi-planar reformatted computed tomography and magnetic resonance images. The blend of anatomical drawings and high-definition medical images helps the reader bridge the void between classroom textbook and radiological anatomy.

The remaining 12 sections form the main body of the publication, including more than 190 interesting cases, with each case demonstrating a particular pathology or condition, and over 1500 quality high-definition images illustrating normal and pathological findings.

Each section is categorized by pathological disorders and covers a vast spectrum of common conditions encountered in day-to-day practice. Section topics range from development abnormalities to thoracic traumas, airways disease to post-chest surgery, and occupational lung pathologies to cardiovascular conditions.

Throughout the atlas a variety of case conditions are reviewed, including atelectasis, pulmonary infections, diffuse lung diseases, congenital lesions, malignancies, and much, much more, which are too numerous to mention in this review.

As mentioned, the overall format of the atlas is basically the same as the first edition with very little change; however, in this second edition we see the introduction of a new post-thoracotomy chest section with additions and expansions to the neoplastic, cardiovascular, and diffuse lung disease sections. Previous case images published in the first edition have also been improved and updated and an extra 40 new interesting cases added. Furthermore, this edition comes with free bonus access to “RadCases online,” which is an online link allowing the reader to access and review an additional 250 thoracic imaging cases.

In writing this atlas the authors have adopted a formulated “case-base-review” programme that was originally developed for and made famous by their lectures at international educational courses and teaching seminars. In short, the programme is designed to replicate everyday radiological practice using an enhanced teaching approach to convey medical knowledge and expertise in a clinically relevant format, placing the student in a theoretical scenario. This approach certainly becomes apparent as the reader starts to progress through the cases.

Each section begins with a well-researched overview of the topic reviewed, expanding on the pathology, aetiology, manifestations, and characteristics of the disease. The authors paint a colourful and informative background picture of the topic in question, leaving nothing to the imagination and providing the reader with an insight of the array of case studies that follow.

The cases are pragmatically organized to simulate actual scenarios following a progressive path covering the initial presentation, radiological findings, and clinical diagnosis. Each case opens with a brief presentation of the patient's clinical history and complaint, giving only the patient's essential details, which would be in keeping with the clinical information provided in clinical practice.

The resultant diagnostic images are presented early in the cases and can be viewed prior to any discussion on radiological findings or clinical diagnosis, allowing the reader to evaluate their radiological knowledge and make an initial clinical assessment.

The radiological findings of the case are presented in a concise, clinical, radiological style report. Clear references are made to the images discussed, and where appropriate, arrows and other symbols are used to draw the reader's attention to areas of radiological and diagnostic significance. The clinical diagnosis is then disclosed with any other differential diagnosis.

Building on the clinical diagnosis, an in-depth discussion then follows, which forms the main part of the case review, providing a detailed background of the particular pathology diagnosed and its aetiology. The reader is then taken on a step-by-step, bullet-point progression of the clinical and image findings, patient management strategies and treatments, and finally concluding with the patient's prognosis.

In most cases major take-home or teaching points which are referred to as “Pearls” are provided to emphasize those features that strongly support a specific diagnosis. The case review concludes with a list of suggested up-to-date readings for those readers keen to expand their clinical knowledge or just interested in a particular case.

In summary, I found the atlas to be an extremely well-researched publication utilizing up-to-date case material, presented in an easy-to-use format from beginning to end. The book covers a vast spectrum of chest conditions, which would unquestionably be encountered in everyday medical practice.

The succinctly and clearly written style combined with the no-nonsense medical vocabulary adopted by the authors is straightforward, and likely to appeal to a wide audience of health professionals. I must mention, however, that the atlas lacks a glossary of medical terminology which would normally accompany publications of this calibre and would undoubtedly be of benefit to the reader by avoiding tedious, time-consuming, cross-referencing.

Upon reading the first section I found it to be highly educational and strongly recommend that the reader take their time to read and absorb this section thoroughly, regardless of their prior knowledge, as the authors have assumed the reader possesses an adequate level of foundation knowledge. This will prove to be good revision for the expert and highly beneficial for the student, making the cases easier to understand and a more enjoyable and effective learning experience.

The case studies are superbly organized and consistent in structure, engaging the reader from the initial presentation. Appropriate key heading and bullet points help the reader to navigate through the cases creating virtual scenarios mimicking clinical practice. Personally, I found that once I completed one case review I was compelled to turn the page and begin the next.

Although an excellent learning and reference resource for the majority of health professionals, I would not recommend this atlas as a replacement radiological teaching manual for chest imaging interpretation as radiological fundamentals and radiological pitfalls are not discussed.

In my opinion I would highly recommend this publication as an invaluable learning tool for radiographers, interns, junior doctors, registrars, trauma and radiology practitioners, and pulmonary–thoracic physicians in particular. Allied health professionals such as nurses and physiotherapists working in the field of thoracic medicine may also find the atlas of great interest.

If the reader gives the book the time it deserves and goes the distance, they will find it greatly enhances and consolidates their clinical practice or alternatively as an ideal comprehensive reference book that can go the rounds and is a champion contender to win a place on any library shelf.

